# Heterogeneous stock market responses to pharmaceutical policy events across subsectors and time scales: Evidence from China

**DOI:** 10.1371/journal.pone.0353989

**Published:** 2026-09-25

**Authors:** Xiangfei Li, Yidan Zhao

**Affiliations:** School of Economics and Management, Tiangong University, Tianjin, China; Universiti Malaysia Sabah, MALAYSIA

## Abstract

Whether abnormal returns around different pharmaceutical policy events exhibit similar patterns, and whether these patterns vary across industry-index levels, return scales, and event windows, has received limited comparative examination within a unified analytical framework. This study examines six pharmaceutical policy events in China using the Shenwan first-level Pharmaceutical and Biotechnology Index and six Shenwan second-level pharmaceutical sub-sector indices, with the CSI 300 Index as the market benchmark. Ensemble Empirical Mode Decomposition (EEMD) is used to reconstruct returns at multiple scales, and an event-study framework is applied to estimate abnormal returns (ARs) and cumulative abnormal returns (CARs). The results show that, within the events, industry indices, and event windows examined, policy objectives or policy-instrument attributes do not exhibit a consistent correspondence with observed abnormal-return patterns. Medium-frequency CARs were generally negative for both centralized procurement events, but centralized drug procurement showed broader negative accumulation across sub-sectors, whereas centralized procurement of high-value medical consumables showed greater sectoral differentiation. Although the clinical trial data self-inspection and verification event and the reform of the drug review and approval system both involved drug R&D and registration regulation, their abnormal-return patterns differed markedly, with the latter showing broadly positive medium-frequency CARs. The aggregate industry index may not fully capture underlying differences when sub-sector responses diverge substantially, and high- and medium-frequency CARs also differ in direction for some events and sub-sectors. In addition, the cumulative direction of CARs over wider event windows may differ from the trajectory of daily ARs following the policy announcement. Overall, generalizations based on policy categories, an aggregate industry index, or a single event window may overlook important differences in abnormal returns across industry-index levels, return scales, and temporal paths.

## Introduction

In China, major pharmaceutical policies are not only part of the institutional environment in which the industry operates, but also important information that capital markets may interpret directly. Market access, health insurance reimbursement, procurement opportunities, price formation, and returns to innovation in pharmaceutical firms are closely related to policy arrangements. Policy changes may therefore alter investors’ expectations about the prospects of related sectors. In this context, it is not unusual for pharmaceutical policies to enter capital market pricing and generate price reactions. The key question is how market reactions differ across pharmaceutical policy events and whether these observed differences show any consistent relationship with policy objectives or policy instrument attributes.

This question is difficult to assess directly. Centralized procurement, price regulation, and innovation-support policies affect different aspects of pharmaceutical firms’ operating environments, while their implications for market valuation may vary across policy contexts and industry segments. Clinical trial data verification, review and approval reform, and DRG/DIP payment reform also contain multiple implications, including regulation, constraint, and institutional adjustment, making their market effects less straightforward. The pharmaceutical sector is also heterogeneous. Chemical pharmaceuticals, traditional Chinese medicine, biological products, pharmaceutical distribution, medical devices, and healthcare services differ substantially in business models, price sensitivity, dependence on medical insurance, and innovation characteristics. As a result, the same policy may generate different reactions across pharmaceutical sub-sector indices. Based on this feature, this study focuses on differences in index-level market reactions across pharmaceutical policy events and examines how these differences are distributed across pharmaceutical sub-sector indices.

Existing research provides a theoretical and methodological basis for examining policy shocks through capital market reactions. According to the efficient market hypothesis, public information can be reflected in asset prices, changes in securities prices can therefore be used to observe market responses to specific information events [1]. Policy announcements and institutional changes may also alter investors’ assessments of future cash flows, risk levels, and industry prospects. Related studies have provided evidence on this issue from the perspective of economic policy uncertainty and its relationship with stock market returns [2,3]. Within this research tradition, event study methods have been widely used to identify the market effects of announcements, regulatory changes, and other information events [4,5] However, information may not be incorporated into prices immediately. Differences in investor attention, information diffusion, and trading behavior can lead to gradual market adjustment [6,7]. Therefore, analyses of capital market reactions to policy shocks should consider not only immediate price changes around the announcement date, but also how abnormal returns are distributed over broader event windows. However, broader symmetric event windows also include pre-event price movements and therefore describe abnormal return patterns around the event rather than a purely post-announcement adjustment process.

Empirical evidence from the pharmaceutical sector further shows that industry-related information can generate identifiable market reactions. Clinical trial results affect the stock prices of biopharmaceutical firms, and positive and negative results are associated with different directions and magnitudes of market response [8,9]. Regulatory approval announcements can also alter investors’ assessments of product prospects and risk exposure [10]. Other types of information, including mergers and acquisitions, R&D progress, regulatory news, and adverse events, may correspond to different abnormal return patterns [11]. In the Chinese context, healthcare fiscal expenditure has been linked to the performance of the pharmaceutical sector index [12]. Centralized procurement policies have been shown to affect R&D investment by pharmaceutical firms [13,14], as well as corporate innovation transformation and financial performance [15,16]. However, existing studies have mainly focused on individual policies, single events, or average firm-level effects. Direct comparisons of market reactions across different types of major pharmaceutical policies remain limited, and few studies have examined whether these reactions are consistent between the pharmaceutical sector index and its sub-sector indices. In addition, most related event studies are based on undecomposed daily returns and prespecified event windows, which does not distinguish whether abnormal returns observed around an event are concentrated in faster- or slower-varying return components.

Against this background, this study examines six major pharmaceutical policy events in China and compares capital market reactions using a pharmaceutical sector index and six pharmaceutical sub-sector indices. To examine these patterns at different return scales, we incorporate EEMD-based scale reconstruction into an event study framework and analyze abnormal returns (ARs) and cumulative abnormal returns (CARs) [17,18]. The analysis focuses on three aspects: first, differences in the direction, magnitude, and industry coverage of abnormal returns across policy events and whether these patterns show any stable correspondence with policy objectives or policy instrument attributes; second, similarities and differences between the overall pharmaceutical sector index and its sub-sector indices; and third, variation in abnormal return patterns across high- and medium-frequency return components, event windows, and daily AR paths.

## Materials and methods

### Study scope, events, and data

This study examines the Chinese pharmaceutical sector index and its main sub-sector indices. The sample includes the Shenwan first-level Pharmaceutical and Biotechnology Index and six Shenwan second-level pharmaceutical sub-sector indices: chemical pharmaceuticals, traditional Chinese medicine, biological products, pharmaceutical distribution, medical devices, and healthcare services. To compare the performance of the pharmaceutical sector and its sub-sectors relative to the overall market, the CSI 300 Index was used as the market benchmark.

Based on the main institutional areas addressed by the pharmaceutical policies discussed above, the comparative analysis covers several policy contexts, including clinical trial regulation, review and approval and market access, procurement and price governance, health insurance payment, and innovation support. Within these contexts, we select national-level policy events with clearly identifiable dates of official public disclosure and substantial institutional significance. The selected events cover different institutional areas while also including policies that use similar instruments but act directly on different targets, thereby providing a basis for comparing abnormal-return patterns across different policy contexts and between events involving similar policy instruments.

Specifically, previous research has regarded the 2015 self-inspection and verification of drug clinical trial data as an important milestone in strengthening clinical trial quality and accelerating drug review and approval in China [19]. The 2017 reform of the drug and medical device review and approval system subsequently set out a systematic series of institutional arrangements for regulatory reform [20]. The “4+7” city pilot represented the first round of state-organized centralized volume-based drug procurement and marked the starting point of the subsequent national procurement system [16], while coronary stents were the first product category covered by state-organized centralized volume-based procurement of high-value medical consumables [21]. Building on earlier pilot programs, the 2021 Three-Year Action Plan for DRG/DIP further expanded payment reform and set the goal of extending DRG/DIP coverage to all eligible medical institutions providing inpatient services by the end of 2025 [22]. The 2025 policy supporting innovative drugs represented a major national-level policy initiative aimed at supporting pharmaceutical innovation. Centralized drug procurement and centralized procurement of high-value medical consumables were both included because they employ similar procurement-governance instruments but apply directly to different product categories. The six events do not constitute a random sample of all pharmaceutical policies implemented during the study period, rather, they are selected cases for comparison across policy contexts. Accordingly, this study does not estimate a general average effect of pharmaceutical policies, and its conclusions are limited to the events, industry indices, return scales, and event windows examined.

Event dates were identified primarily according to the release of new information that could be incorporated into market prices. For formal policy documents, the official public release date was used as the policy information date. For centralized procurement events, we used the date on which the provisional selection results were officially announced, because key information, including the provisionally selected firms, proposed selection prices, and procurement scope, was disclosed at that point. This date therefore more closely represents the information event at which capital-market participants could reassess expectations for the affected industries. If the policy information date fell on a trading day, that day was defined as the event date (t = 0); if it fell on a non-trading day, the next available trading day was used. The selected events and corresponding dates are reported in Table 1.

**Table 1:** Selected major pharmaceutical policy events.

No.	Policy event	Policy instrument and institutional area	Policy information date	Event date
1	Announcement by the China Food and Drug Administration on self-inspection and verification of drug clinical trial data	Regulation of clinical trial data quality and compliance in registration submissions	2015-07-22	2015-07-22
2	Issuance of the Opinions on Deepening the Reform of the Review and Approval System and Encouraging Innovation in Drugs and Medical Devices by the General Office of the CPC Central Committee and the General Office of the State Council	Review and approval and market access	2017-10-08	2017-10-09
3	Formal disclosure of the proposed selected results of the “4+7” pilot city centralized drug procurement program	Drug procurement and price governance	2018-12-07	2018-12-07
4	Release of the proposed selected results of the first national centralized volume-based procurement of high-value medical consumables	Procurement and price governance of high-value medical consumables	2020-11-05	2020-11-05
5	Issuance of the Three-Year Action Plan for DRG/DIP Payment Reform	Health insurance payment reform	2021-11-26	2021-11-26
6	Issuance of the Several Measures to Support the High-Quality Development of Innovative Drugs by the National Healthcare Security Administration and the National Health Commission	Innovative drug support	2025-07-01	2025-07-01

Source: Compiled by the authors based on publicly available policy documents from the official websites of the China Food and Drug Administration, the General Office of the CPC Central Committee, the General Office of the State Council, the Shanghai Sunshine Pharmaceutical Procurement Network, the National Healthcare Security Administration, and other official sources.

**Fig 1:**
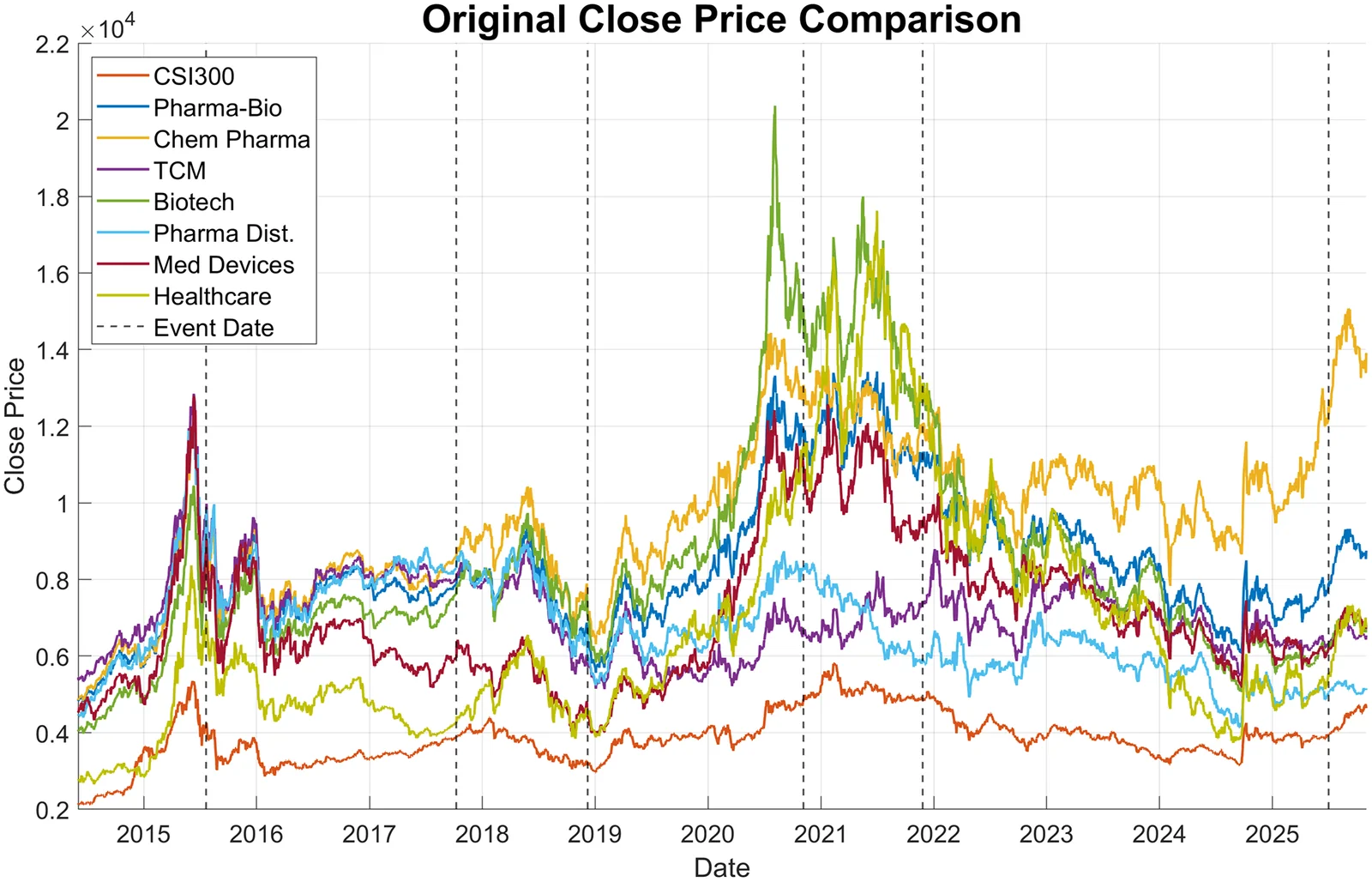
Raw price trends of the study indices.

Daily closing-price data were obtained from the iFinD database for the period from June 4, 2014, to October 31, 2025. All indices were matched by trading date. If any index had a missing observation on a given trading day, that date was excluded from all series, and no interpolation was performed. The original price series are shown in Fig 1.

Daily simple returns were calculated from the closing prices of consecutive valid trading days and used as the basic variables for the subsequent analysis:


$$\begin{array}{c} {\text{R}}_{\text{t}}=\frac{{\text{P}}_{\text{t}}-{\text{P}}_{\text{t}-\text{1}}}{{\text{P}}_{\text{t}-\text{1}}} \end{array}$$ (1)

Here, ${\text{P}}_{\text{t}}$ represents the closing price on the t-th trading day, ${\text{P}}_{\text{t}-\text{1}}$ represents the closing price on the previous trading day, and ${\text{R}}_{\text{t}}$ represents the simple return on the t-th trading day.

### EEMD decomposition and scale reconstruction

To obtain return components at different frequency scales, we applied ensemble empirical mode decomposition (EEMD) separately to the return series of the CSI 300 Index, the Shenwan first-level Pharmaceutical and Biotechnology Index, and the six Shenwan second-level pharmaceutical sub-sector indices. EEMD helps mitigate the mode-mixing problem that may arise from a single EMD by repeatedly adding independent white noise to the original series, performing empirical mode decomposition on each noise-added series, and then ensemble-averaging the corresponding intrinsic mode functions (IMFs) [17].

Let the original return series be ${\text{r}}_{\text{n}}\left(\text{t}\right)$, then the EEMD decomposition result can be expressed as:


$$\begin{array}{c} \text{X}\left(\text{t}\right)=\sum_{\text{j}=\text{1}}^{\text{n}}{\text{IMF}}_{\text{j}}\left(\text{t}\right)+{\text{r}}_{\text{n}}\left(\text{t}\right) \end{array}$$ (2)

Here, ${\text{IMF}}_{\text{j}}$ denotes the j-th IMF component, and ${\text{r}}_{\text{n}}\left(\text{t}\right)$ denotes the residual term.

In the baseline analysis, the EEMD parameters were set to Nstd = 0.20 and NR = 100, with a maximum of 1000 sifting iterations and a fixed random seed. EEMD was applied to the full-sample return series. The resulting frequency components were therefore used for ex post characterization of the multiscale structure of returns around the policy events, rather than for constructing real-time trading signals or identifying causal effects of the policies.

EMD exhibits an approximate filter-bank property when applied to stochastic signals, with the characteristic periods of successive intrinsic mode functions (IMFs) generally increasing with IMF order [23]. Based on the progressive increase in the average periods of the IMFs, together with their standard deviations and proportions of variance, IMF1–IMF3 were reconstructed as the high-frequency component, IMF4–IMF6 as the medium-frequency component, and IMF7 and subsequent IMFs, together with the residual, as the low-frequency component. The scale-reconstruction scheme is presented in Table 2, and complete IMF statistics are provided in the Supporting Information. The low-frequency component primarily captures gradual changes over longer time scales. Within the relatively short event windows examined in this study, such variation is difficult to attribute specifically to abnormal returns around a single policy event and was therefore not a primary focus of the event analysis in the main text.

**Table 2:** EEMD-based scale reconstruction rules.

Scale	IMF components	Interpretation
High-frequency component	IMF1—IMF3	Return fluctuations over shorter time scales
Medium-frequency component	IMF4—IMF6	Return fluctuations over intermediate time scales
Low-frequency component	IMF7—IMF-end + Residual	Changes and trends over longer time scales

### Event study design

After EEMD decomposition and scale reconstruction, this study used an event study framework to examine the market reactions of the pharmaceutical sector index and its sub-sector indices to major pharmaceutical policy events. Previous studies have shown that daily returns are suitable for short-window event studies and that short event windows are better suited to identifying market reactions to information events [4,5]. Accordingly, normal returns were estimated and abnormal returns were calculated separately using high-frequency and medium-frequency reconstructed returns, allowing comparison of market reactions to policy shocks across different time scales.

The event dates listed in Table 1 were defined as t = 0. The estimation window was set from the 260th to the 11th trading day before the event, i.e., [−260, −11], to estimate the normal return model while avoiding overlap with the event windows. Daily abnormal returns (ARs) were examined over the interval [−10, +20]. Cumulative abnormal returns (CARs) were calculated over four symmetric event windows: [−1, +1], [−3, +3], [−5, +5], and [−10, +10]. Because all of these windows include pre-event trading days, CARs over the wider windows should be interpreted together with the daily AR trajectories and should not be attributed entirely to post-announcement price adjustment.

This study used the market model to estimate normal returns. For the reconstructed return of index i on day t, the market model was specified as:


$$\begin{array}{c} {\text{R}}_{\text{i},\text{t}}=\alpha_{\text{i}}+\beta_{\text{i}}{\text{R}}_{\text{m},\text{t}}+\epsilon_{\text{i},\text{t}} \end{array}$$ (3)

Here, ${\text{R}}_{\text{i},\text{t}}$ denotes the reconstructed return of industry i on day t, ${\text{R}}_{\text{m},\text{t}}$ denotes the reconstructed return of the CSI 300 Index on day t, $\alpha_{\text{i}}$ and $\beta_{\text{i}}$ are parameters estimated using the least squares method within the estimation window, while $\epsilon_{\text{i},\text{t}}$ is the residual term.

An abnormal return was defined as the difference between the actual return of an industry index on a given trading day at a given scale and the normal return estimated from the market model. The cumulative abnormal return was defined as the sum of abnormal returns within a given event window, that is, the sum of abnormal returns across all trading days in that window.

### Sensitivity analysis design

To assess whether the main medium-frequency CAR results depend on specific EEMD settings, we examined the sensitivity of the results to both IMF grouping boundaries and EEMD parameters. The sensitivity analysis focused on the medium-frequency component because the principal cross-event and cross-sector comparisons in this study are primarily based on medium-frequency CAR patterns, while the high-frequency results are used to characterize differences in abnormal-return patterns across statistical scales.

For the grouping sensitivity analysis, the original EEMD decomposition was retained, and four alternative grouping specifications were examined separately: IMF3 was reassigned to the medium-frequency component, IMF4 to the high-frequency component, IMF6 to the low-frequency component, and IMF7 to the medium-frequency component. Returns at each frequency scale were then reconstructed accordingly. For the parameter sensitivity analysis, the baseline IMF grouping was retained, while Nstd was separately changed from 0.20 to 0.10 and 0.30 and NR from 100 to 50 and 200, with all other parameters held at their baseline values. EEMD was rerun under each alternative parameter setting, with the maximum number of sifting iterations fixed at 1000. Under all alternative specifications, ARs and CARs were recalculated using the same event dates, estimation window, event windows, market benchmark, and market model as in the baseline analysis.

Sensitivity was evaluated using the 168 medium-frequency CARs generated by the combination of six events, seven industry indices, and four event windows. For the IMF grouping sensitivity analysis, comparisons focused on direction, relative ranking, and magnitude. The directional agreement rate was defined as the proportion of event-index-window combinations for which the CAR had the same sign under an alternative grouping as under the baseline grouping. Spearman’s rank correlation coefficient was used to assess the consistency of CAR rankings between each alternative grouping and the baseline grouping, while changes in magnitude were measured using the median absolute difference between the alternative and baseline CARs. For the EEMD parameter sensitivity analysis, comparisons focused on direction and magnitude. Directional agreement was calculated in the same manner, and changes in magnitude were summarized using both the mean and median absolute differences between the CARs obtained under each alternative parameter setting and the corresponding baseline CARs.

## Results

### Medium-frequency return paths across policy events

**Fig 2:**
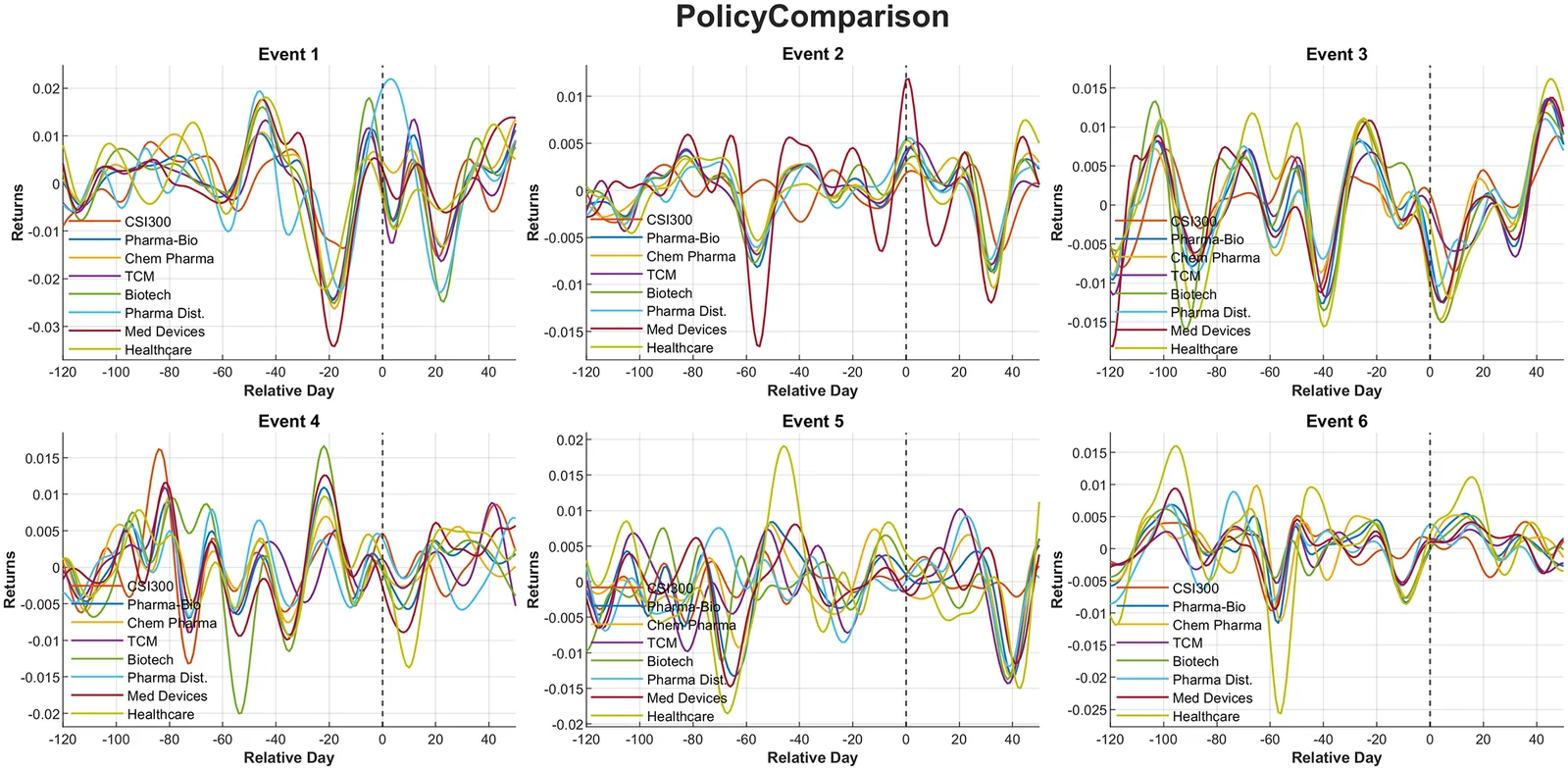
Evolution of medium-frequency returns across the pharmaceutical sector and sub-sector indices.

Fig 2 shows the reconstructed medium-frequency return paths of the CSI 300 Index, the Shenwan first-level Pharmaceutical and Biotechnology Index, and six Shenwan second-level pharmaceutical sub-sector indices around the six policy events. Because these returns have not been adjusted for movements in the market benchmark, the figure is used primarily to characterize the medium-frequency return patterns around the event dates and to compare differences across events and industry indices.

In Event 1, the Pharmaceutical Distribution Index showed the most pronounced movement. It remained at a relatively high level around the event date, continued to rise briefly after the event, and then declined rapidly, whereas most other pharmaceutical indices initially fell and subsequently recovered. In Event 2, the Medical Devices Index reached a distinct short-term peak around the event date and then declined. Its overall direction was broadly similar to that of most pharmaceutical sub-sector indices, but the magnitude of its fluctuations was greater. Events 3 and 4 both showed relatively pronounced downward movements. In Event 3, most pharmaceutical indices moved into negative territory around the event date and remained at relatively low levels for a short period afterward, although the depth of the decline and the pace of recovery varied across sub-sectors. In Event 4, most indices weakened further after the event, with particularly pronounced declines in Healthcare Services and Medical Devices. By contrast, medium-frequency movements around Events 5 and 6 were relatively moderate. In Event 5, most pharmaceutical indices fluctuated within a narrow range around zero, without a clear common upward or downward pattern. In Event 6, most pharmaceutical sub-sector indices recovered from negative values before the event date to around zero or slightly positive values, but movements around the event date were limited and no sustained common upward trend emerged afterward.

Overall, medium-frequency return paths around the six policy events differed in direction, magnitude, and the degree of co-movement across industry indices. Because Fig 2 presents returns before adjustment for the market benchmark, these patterns should not be interpreted directly as abnormal returns. The following analysis therefore compares high- and medium-frequency ARs and CARs across different event windows.

### Differences in abnormal returns across policy events

The following analysis compares high- and medium-frequency ARs and CARs across different event windows for the six events, organized into three policy domains: centralized procurement, clinical trial regulation and review and approval reform, and payment reform and innovation support. The comparison focuses on the direction and sectoral coverage of abnormal returns, as well as differences across return scales and event windows. Because CARs are calculated over symmetric windows around the event date, they incorporate abnormal returns both before and after the event. Daily ARs are therefore also examined to assess the temporal composition of CARs across different windows.

**Fig 3:**
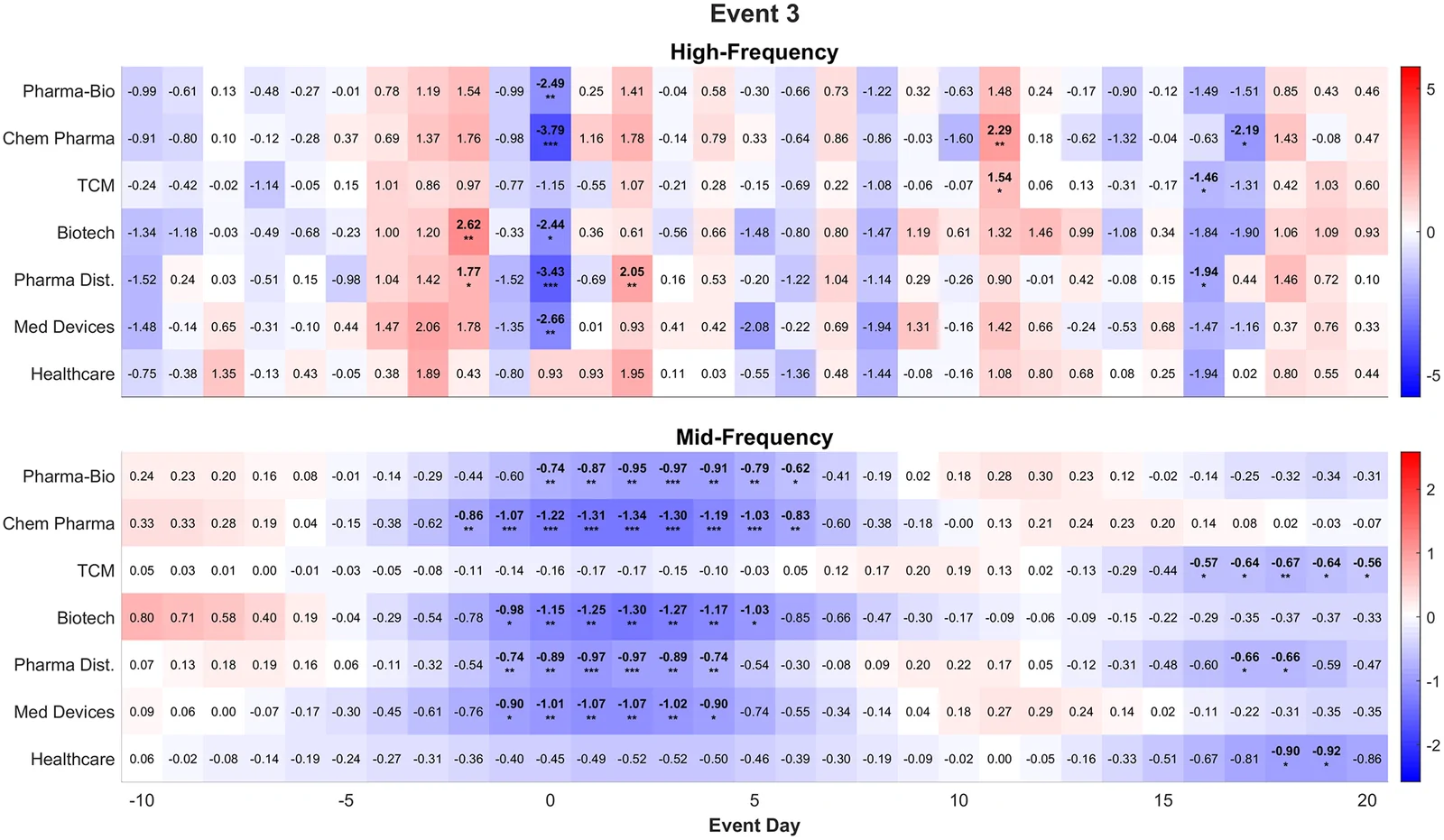
High- and medium-frequency AR heatmaps for Event 3 (%).

Medium-frequency CARs were generally negative for both centralized procurement events, but the distribution of these negative returns across sub-sectors differed. As shown in Table 3, the “4+7” centralized drug procurement event (Event 3) showed broad negative accumulation across sub-sectors. The Shenwan first-level Pharmaceutical and Biotechnology Index and all six pharmaceutical sub-sector indices had negative CARs in all four medium-frequency event windows. Chemical Pharmaceuticals showed the largest negative magnitude, with a CAR of −11.29% in the [−10, +10] window. Fig 3 also shows that negative medium-frequency ARs had already emerged in several sub-sectors before the event date and persisted for some time afterward. This broad cross-sub-sector consistency was not observed at the high-frequency scale. For example, in the [−5, +5] window, the high-frequency CARs of all seven indices were positive, in marked contrast to the uniformly negative medium-frequency CARs over the same window.

**Table 3:** High- and medium-frequency CAR results for Events 3 and 4 (%).

NO.	Index	High frequency				Medium frequency			
		[−1,1]	[−3,3]	[−5,5]	[−10,10]	[−1,1]	[−3,3]	[−5,5]	[−10,10]
3	Pharma-Bio	−3.22*	0.88	1.94	−1.75	−2.21***	−4.85***	−6.71***	−6.82***
	Chem Pharm	−3.60*	1.17	3.35	−0.93	−3.60***	−7.72***	−10.47***	−11.29***
	TCM	−2.47*	0.23	1.52	−2.04	−0.47	−0.97	−1.17	−0.36
	Biotech	−2.41	1.46	1.41	−1.99	−3.39***	−7.26***	−9.80***	−9.57***
	Pharma Dist.	−5.64***	−0.24	0.15	−2.75	−2.61***	−5.33***	−6.65***	−5.79***
	Med Devices	−4.00*	1.19	1.45	−0.24	−2.98***	−6.44***	−8.83***	−9.73***
	Healthcare	1.06	5.44	5.26	3.20	−1.35	−3.06**	−4.53**	−5.88**
4	Pharma-Bio	−2.43	−2.41	−2.08	−3.28	−0.66	−1.28	−1.68	−4.04**
	Chem Pharm	−0.29	0.33	−0.91	2.49	−0.72	−1.54	−2.21*	−4.38***
	TCM	−0.79	−0.98	−1.31	−2.82	−0.18	−0.56	−1.22	−3.15**
	Biotech	−4.00	−3.27	0.32	−3.59	−0.66	−1.57	−2.57	−6.02**
	Pharma Dist.	−1.63	−2.85	0.36	−2.48	1.24	2.58**	3.31**	2.07
	Med Devices	−5.19*	−4.63	−3.55	−3.94	−1.20	−2.64**	−3.86**	−7.15***
	Healthcare	−2.32	−2.36	−2.06	−3.74	−0.74	−1.42	−1.89	−4.93**

Note: *, **, and *** indicate significance at the 10%, 5%, and 1% levels, respectively.

**Fig 4:**
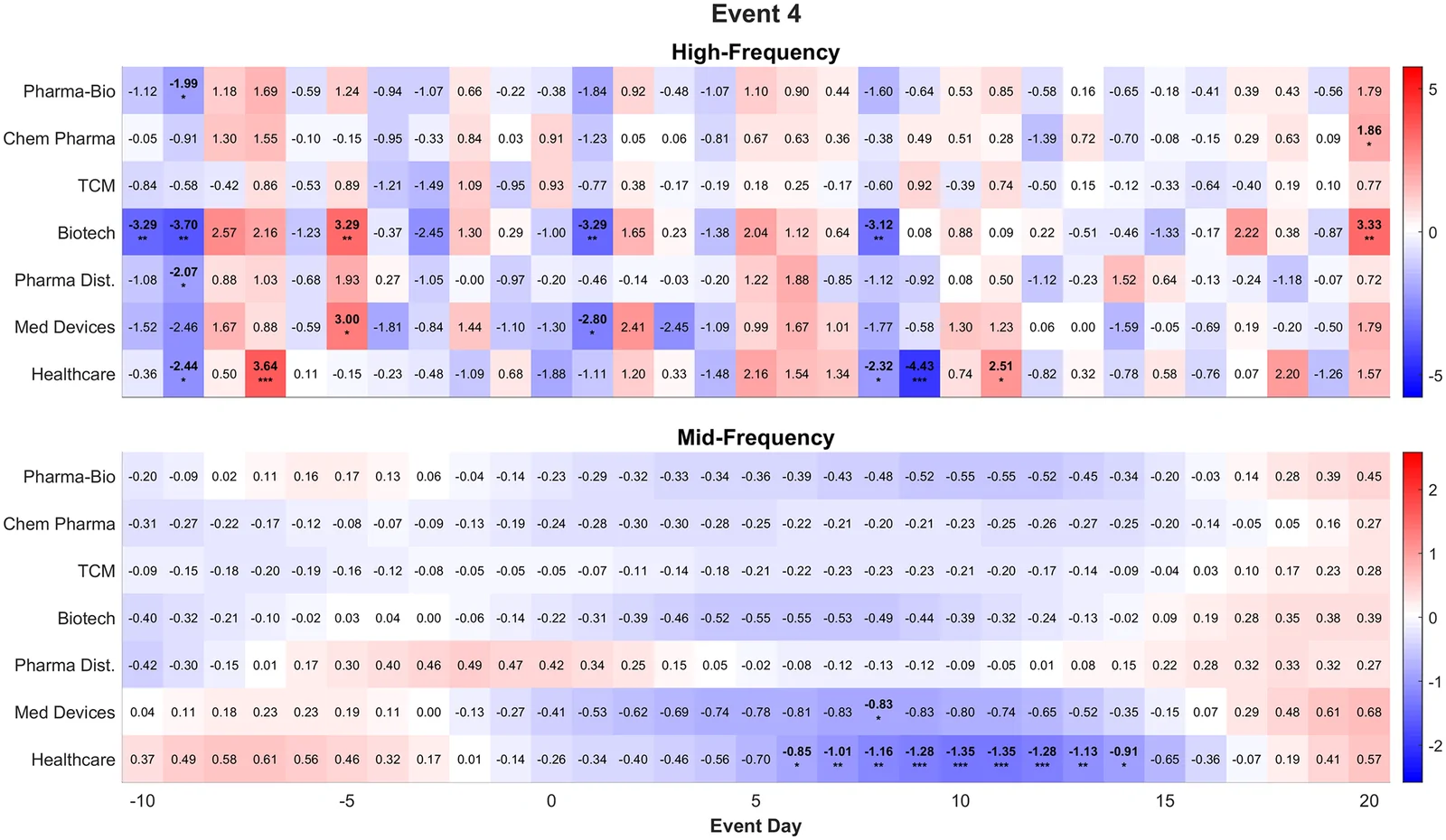
High- and medium-frequency AR heatmaps for Event 4 (%).

The centralized procurement of high-value medical consumables (Event 4) was likewise characterized mainly by negative medium-frequency CARs, but with greater sectoral differentiation. Except for Pharmaceutical Distribution, all indices had negative CARs across all four medium-frequency windows, whereas Pharmaceutical Distribution remained positive throughout. Medical Devices showed the largest negative magnitude, with a medium-frequency CAR of −7.15% in the [−10, +10] window; its CARs were also the lowest among the seven indices in all four high-frequency windows. Fig 4 further shows relatively persistent negative medium-frequency ARs for Medical Devices around the event date. Overall, Event 3 was characterized by broad negative accumulation across sub-sectors, whereas Event 4 showed more pronounced sectoral differentiation against a generally negative medium-frequency pattern.

**Fig 5:**
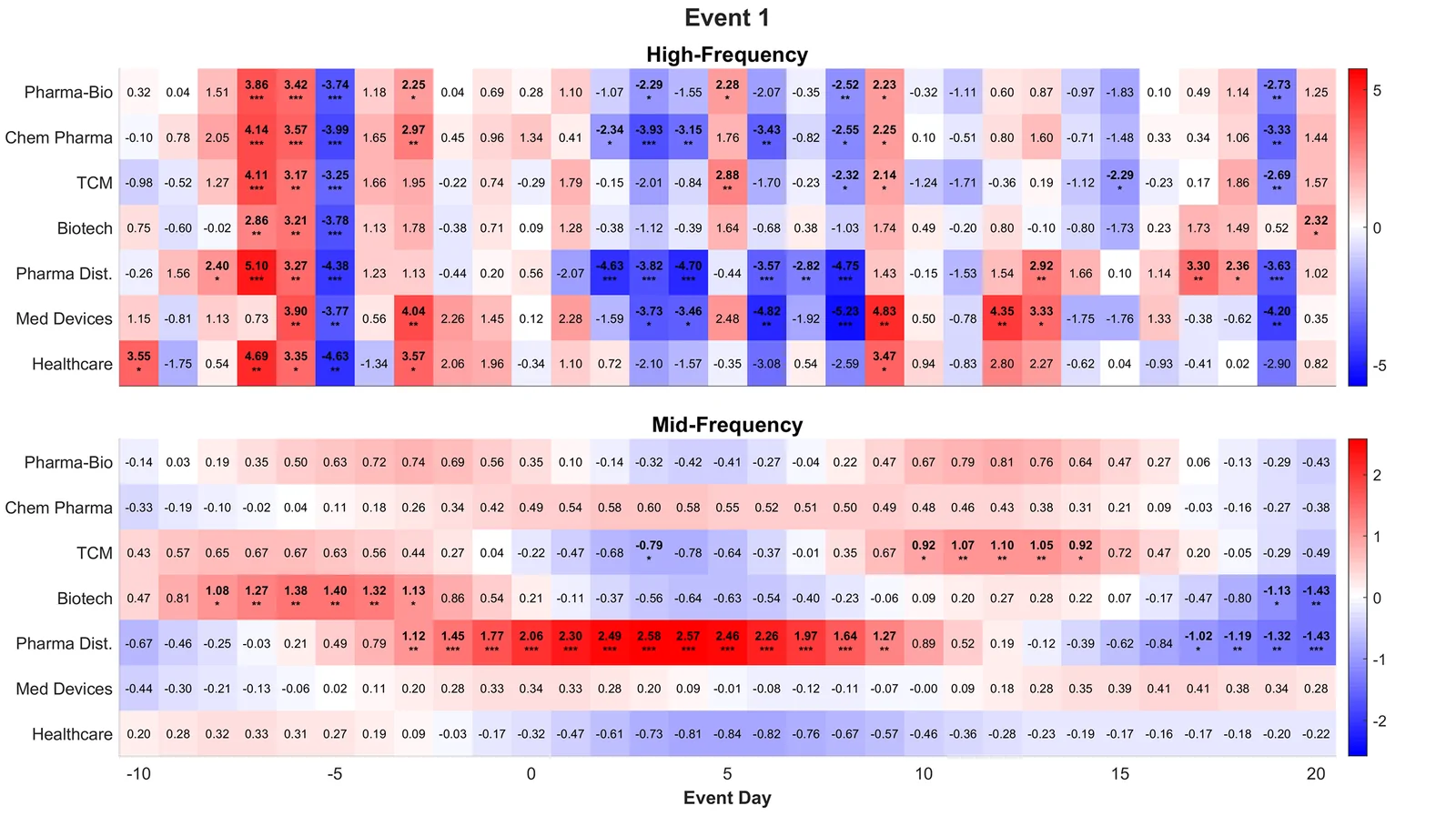
High- and medium-frequency AR heatmaps for Event 1 (%).

The clinical trial data self-inspection and verification event (Event 1) and the review and approval system reform (Event 2) exhibited distinct abnormal-return patterns. Event 1 was characterized primarily by marked differences across pharmaceutical sub-sectors and between high- and medium-frequency return scales. Table 4 shows that the Shenwan first-level Pharmaceutical and Biotechnology Index had positive CARs across all four medium-frequency windows, whereas the pharmaceutical sub-sector indices did not show a consistent direction. Healthcare Services, for example, had negative CARs in all four windows. The contrast across return scales was most pronounced for Pharmaceutical Distribution: its CARs were negative in all four high-frequency windows but positive in all four medium-frequency windows, reaching −15.15% and 26.90%, respectively, in the [−10, + 10] window. Fig 5 further shows that positive medium-frequency ARs for Pharmaceutical Distribution had already emerged before the event date and persisted for some time afterward. The positive CAR over the wider window therefore reflected the accumulation of abnormal returns both before and after the event.

**Table 4:** High- and medium-frequency CAR results for Events 1 and 2 (%).

NO.	Index	High frequency				Medium frequency			
		[−1,1]	[−3,3]	[−5,5]	[−10,10]	[−1,1]	[−3,3]	[−5,5]	[−10,10]
1	Pharma-Bio	2.07	0.99	−0.83	5.30	1.02	2.00	2.52	4.52*
	Chem Pharm	2.70	−0.14	−3.87	2.12	1.45	3.23**	4.66**	6.56**
	TCM	2.24	1.80	2.25	5.95	−0.64	−1.40	−1.63	2.93
	Biotech	2.08	1.98	0.58	7.70	0.64	1.69	3.14	7.03**
	Pharma Dist.	−1.30	−9.06**	−17.35***	−15.15**	6.13***	13.76***	20.08***	26.90***
	Med Devices	3.85	4.83	0.65	0.08	1.00	1.96	2.18	0.66
	Healthcare	2.72	6.97	−0.92	8.73	−0.95	−2.22	−3.42	−5.27
2	Pharma-Bio	1.47*	0.87	−0.38	0.88	0.90**	1.90***	2.54***	2.66**
	Chem Pharm	0.83	1.52	0.31	0.93	0.50	1.05*	1.39*	1.56
	TCM	1.04	0.21	−0.43	0.95	0.75*	1.75***	2.67***	4.10***
	Biotech	1.37	1.28	0.24	0.56	0.63	1.40**	2.03**	3.02**
	Pharma Dist.	2.05**	1.85	0.36	3.44	1.41***	3.14***	4.55***	6.31***
	Med Devices	3.81**	0.37	−3.93	−0.78	3.13***	6.15***	7.16***	3.02
	Healthcare	2.61*	−0.39	−2.04	−1.55	1.00**	2.12***	2.85***	3.12***

Note: *, **, and *** indicate significance at the 10%, 5%, and 1% levels, respectively.

**Fig 6:**
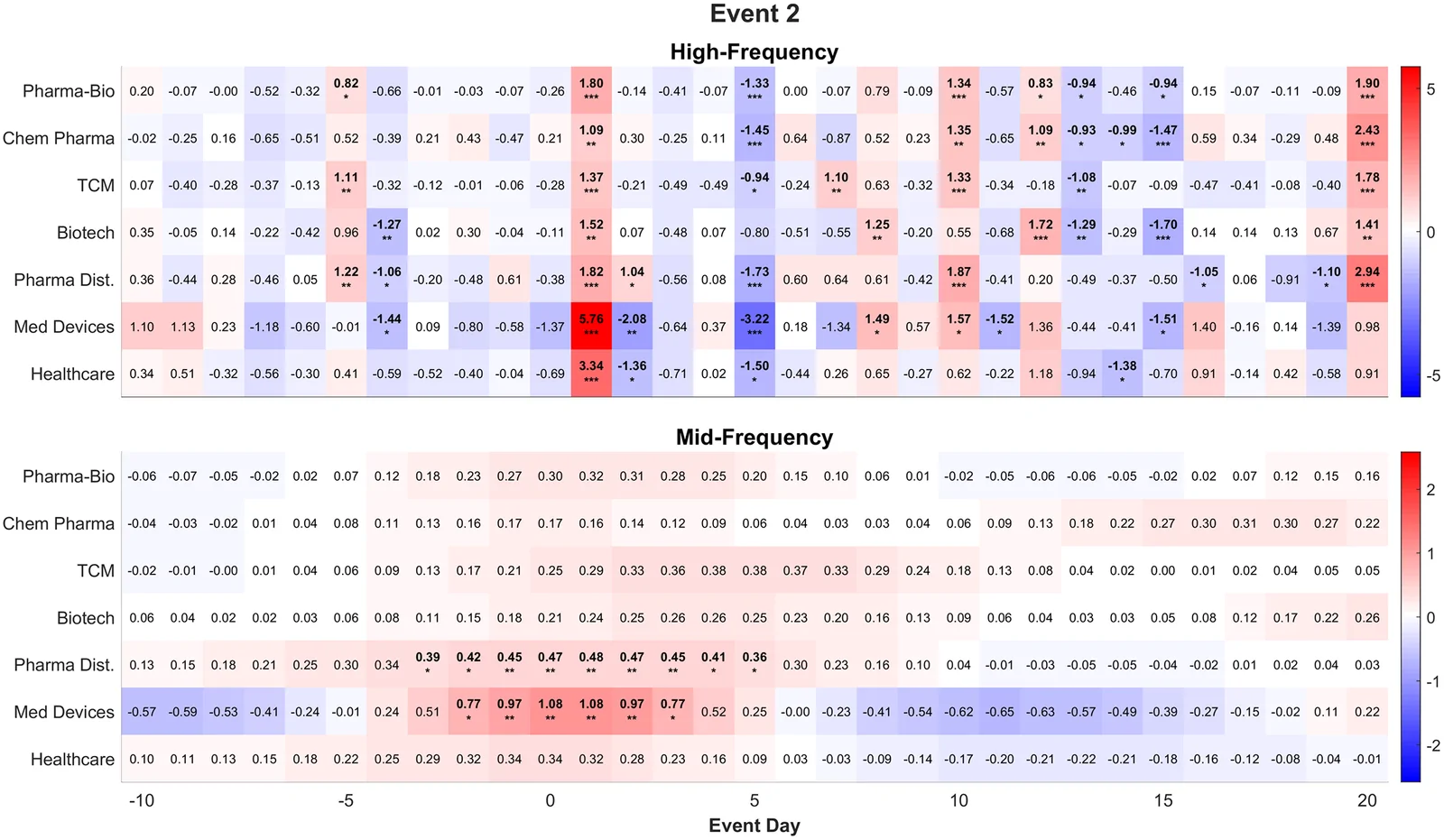
High- and medium-frequency AR heatmaps for Event 2 (%).

By contrast, the medium-frequency results for Event 2 were more consistent across industry indices. The Shenwan first-level Pharmaceutical and Biotechnology Index and all six pharmaceutical sub-sector indices had positive CARs across all four medium-frequency windows, although the magnitude of accumulation varied across windows. For example, the medium-frequency CAR for Medical Devices decreased from 7.16% in the [−5, + 5] window to 3.02% in the [−10, + 10] window, indicating that the stronger positive accumulation was concentrated within the shorter window. Fig 6 also shows that the high-frequency ARs of all seven indices were positive and statistically significant on the first trading day after the event. Positive and negative ARs then alternated again, resulting in greater cross-sector differentiation in high-frequency CARs over the wider windows. Overall, Event 1 was characterized mainly by pronounced differences across sub-sectors and return scales, whereas Event 2 showed broadly positive medium-frequency CARs, although the magnitude and temporal path of accumulation still varied across sub-sectors.

**Fig 7:**
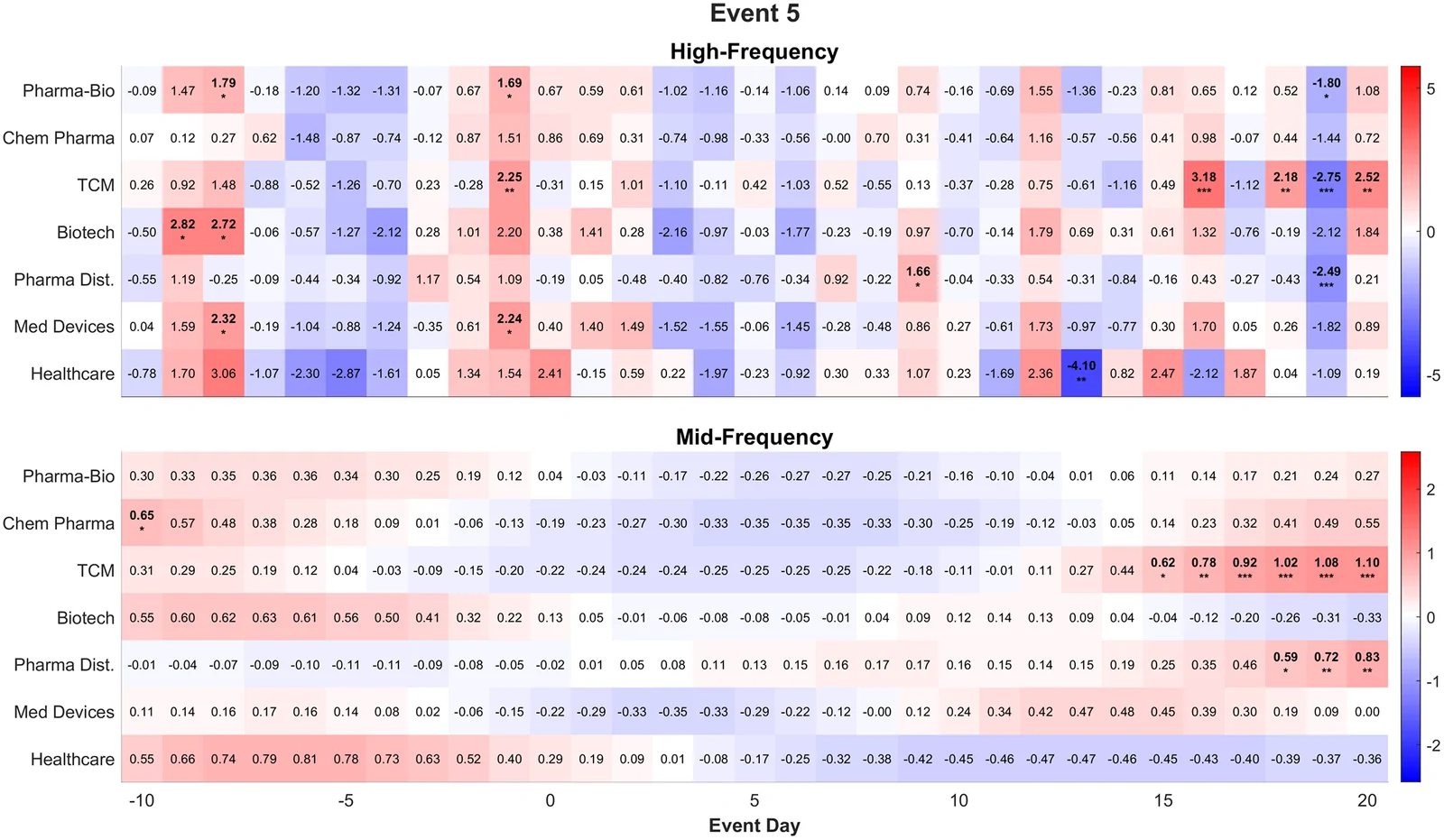
High- and medium-frequency AR heatmaps for Event 5 (%).

The payment reform and innovation support events both showed pronounced differences across return scales and sub-sectors, although the specific patterns differed. Event 5 was characterized primarily by broadly positive high-frequency CARs over shorter event windows alongside substantial cross-sector differentiation at the medium-frequency scale. Table 5 shows that the high-frequency CARs of all seven indices were positive in the [−1, + 1] and [−3, + 3] windows. At the medium-frequency scale, however, Chemical Pharmaceuticals, Traditional Chinese Medicine, and Medical Devices had negative CARs across all four windows, whereas the Shenwan first-level Pharmaceutical and Biotechnology Index, Biological Products, and Healthcare Services had positive CARs throughout. Fig 7 further shows that some positive medium-frequency ARs had already emerged before the event date and gradually weakened afterward. CARs over the wider windows therefore incorporated abnormal returns from both the pre- and post-event periods.

**Table 5:** High- and medium-frequency CAR results for Events 5 and 6 (%).

NO.	Index	High frequency				Medium frequency			
		[−1,1]	[−3,3]	[−5,5]	[−10,10]	[−1,1]	[−3,3]	[−5,5]	[−10,10]
5	Pharma-Bio	2.95*	3.15	−0.79	0.77	0.13	0.29	0.46	0.98
	Chem Pharm	3.05*	3.37	0.44	0.08	−0.55	−1.18	−1.57	−0.78
	TCM	2.09	1.97	0.32	0.28	−0.66	−1.39	−1.87*	−1.73
	Biotech	3.99	3.39	−0.99	1.49	0.41	1.07	1.98	5.16**
	Pharma Dist.	0.96	1.79	−1.05	0.79	−0.06	−0.11	−0.08	0.41
	Med Devices	4.04*	4.28	0.54	2.20	−0.66	−1.38	−1.78	−1.03
	Healthcare	3.80	6.00	−0.68	0.95	0.88	2.13	3.39	5.12
6	Pharma-Bio	1.53	2.62	0.28	0.87	−0.49	−1.18	−1.96*	−4.00***
	Chem Pharm	1.18	2.76	−1.63	−2.04	−0.12	−0.33	−0.64	−1.71
	TCM	−0.29	0.68	−0.25	−0.26	0.20	0.37	0.32	−0.81
	Biotech	1.29	3.26	0.27	2.10	−0.49	−1.19	−2.01*	−4.22***
	Pharma Dist.	0.70	1.56	0.78	−0.88	0.84	1.67	1.93	0.47
	Med Devices	1.16	1.53	−0.34	0.15	−0.10	−0.30	−0.64	−1.94
	Healthcare	2.11	0.41	−1.56	−1.43	−0.03	−0.20	−0.63	−1.86

Note: *, **, and *** indicate significance at the 10%, 5%, and 1% levels, respectively.

**Fig 8:**
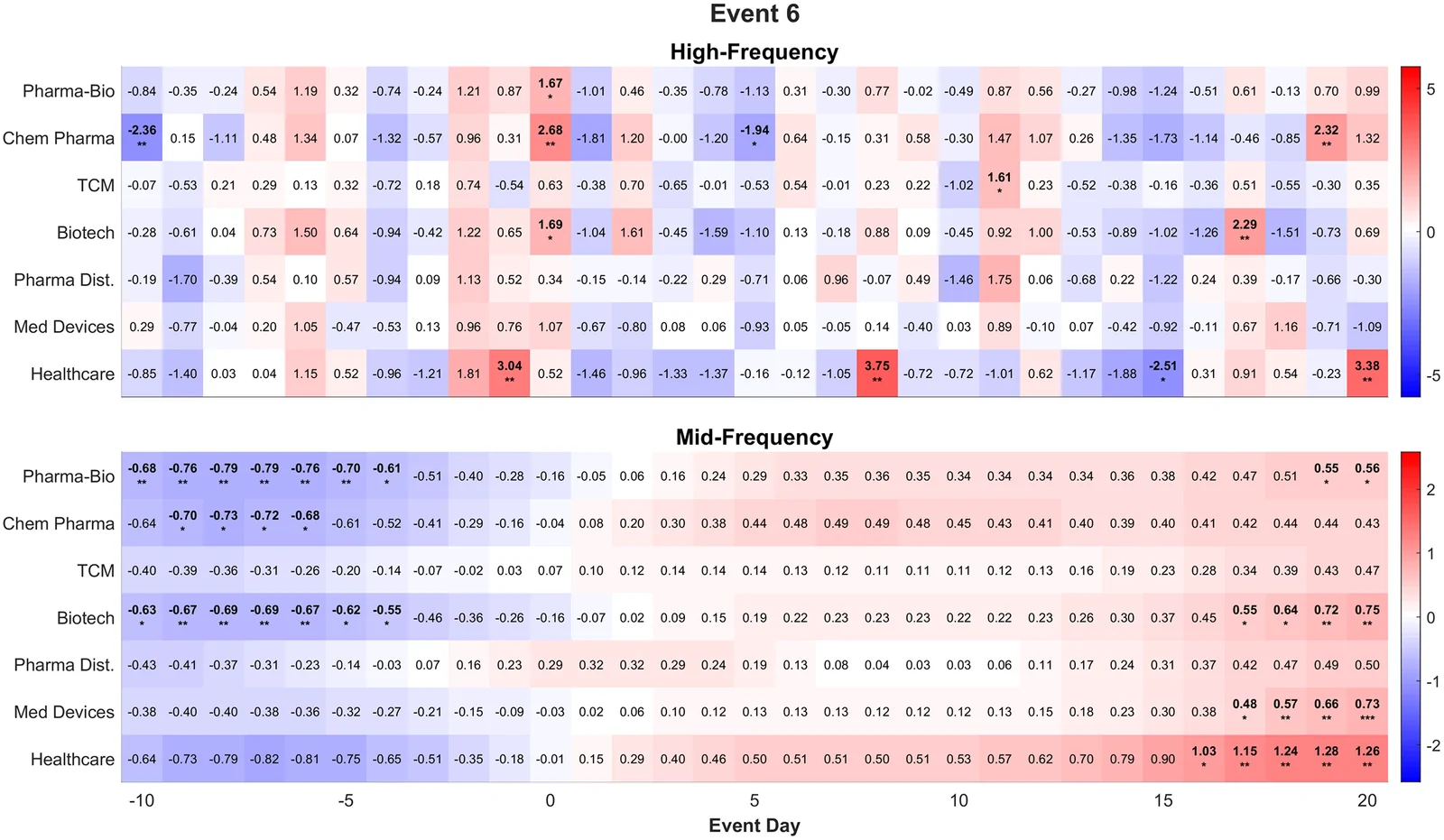
High- and medium-frequency AR heatmaps for Event 6 (%).

Event 6 showed a different temporal pattern. The high-frequency AR on the event date and CARs over the shorter windows were generally positive, but greater differentiation emerged as the event window widened. Medium-frequency CARs, by contrast, were predominantly negative, with the Shenwan first-level Pharmaceutical and Biotechnology Index and Biological Products recording CARs of −4.00% and −4.22%, respectively, in the [−10, + 10] window. However, Fig 8 shows that medium-frequency ARs for most indices were negative before the event date and subsequently increased, with all seven indices turning positive from the second trading day after the event. Thus, Event 5 was characterized more prominently by differences across sub-sectors and return scales, whereas the negative CARs over wider windows in Event 6 were more strongly influenced by negative pre-event ARs. Their cumulative direction therefore did not fully correspond to the trajectory of daily ARs after the event.

Across the six policy events, abnormal returns differed in direction, sectoral coverage, and return scale, and policy objectives or policy-instrument attributes did not show a consistent correspondence with the observed abnormal-return patterns. Both centralized procurement events were characterized mainly by negative medium-frequency CARs, although the breadth of sectoral coverage differed between them, whereas the review and approval system reform showed broadly positive medium-frequency CARs. The same event could also produce different results across the first-level pharmaceutical index and its sub-sector indices and between high- and medium-frequency return components. In addition, some CARs over wider event windows were substantially influenced by abnormal returns that had already emerged before the event date. Overall, specific market responses need to be interpreted in relation to the individual event, industry level, return scale, and temporal path.

### Sensitivity analysis results

The sensitivity results for the IMF grouping boundaries are presented in Table 6. Across the four alternative grouping schemes, the directional consistency rate of medium-frequency CARs relative to the baseline specification ranged from 88.1% to 91.1%, Spearman’s rank correlation coefficient ranged from 0.869 to 0.934, and the median absolute CAR difference ranged from 0.480 to 0.804 percentage points. Reassigning adjacent IMFs changed the magnitude and, in some cases, the sign of individual CARs. Nevertheless, most of the 168 CAR combinations retained the same positive or negative direction as under the baseline specification, and their relative rankings also remained highly consistent. These results indicate that the choice of IMF grouping boundaries affected some individual estimates, but the main differences in medium-frequency CARs across policy events and sub-sectors were not highly dependent on any single grouping scheme.

**Table 6:** Sensitivity analysis of IMF grouping boundaries for medium-frequency CAR results.

Alternative IMF Grouping	Directional Consistency	Spearman’s ρ	Median Absolute CAR Difference (percentage points)
IMF3 → MF	88.1%	0.905	0.782
IMF4 → HF	88.1%	0.880	0.804
IMF6 → LF	91.1%	0.869	0.718
IMF7 → MF	91.1%	0.934	0.480

The EEMD parameter sensitivity results are presented in Table 7. When Nstd was changed from 0.20 to 0.10 or 0.30 and NR from 100 to 50 or 200, the directional consistency rate of medium-frequency CARs exceeded 96% under all alternative parameter settings. The mean absolute CAR difference ranged from 0.188 to 0.453 percentage points, and the median absolute CAR difference ranged from 0.110 to 0.291 percentage points. Compared with changes in the IMF grouping boundaries, changes in the EEMD parameters produced smaller overall differences in CARs, indicating that medium-frequency CARs were relatively insensitive to changes in noise amplitude and ensemble size within the parameter ranges examined in this study.

**Table 7:** Sensitivity analysis of EEMD parameter settings for medium-frequency CAR results.

Parameter Setting	Directional Consistency	Mean Absolute CAR Difference (percentage points)	Median Absolute CAR Difference (percentage points)
Nstd = 0.10	98.2%	0.453	0.291
Nstd = 0.30	96.4%	0.356	0.199
NR = 50	96.4%	0.226	0.161
NR = 200	98.2%	0.188	0.110

Overall, changes in the IMF grouping boundaries had a greater effect on medium-frequency CARs than changes in Nstd and NR. However, under both types of alternative specification, most CARs retained the same direction as in the baseline analysis and the changes in magnitude were limited. The main differences in medium-frequency CARs across policy events and sub-sectors therefore remained broadly stable under the alternative settings examined.

## Discussion

Among the six pharmaceutical policy events examined in this study, the supportive or restrictive orientation of a policy did not consistently correspond to the direction of abnormal returns, and similar policy instruments could be associated with different sectoral response patterns. The “4+7” centralized drug procurement event showed broadly negative medium-frequency CARs, consistent with firm-level studies reporting negative market reactions and substantial heterogeneity across firms [13,24]. This correspondence, however, did not extend across all events. The clinical trial data self-inspection and verification event was not associated with broadly negative medium-frequency CARs, while the innovative drug support event, despite its explicitly supportive orientation, was not associated with broadly and consistently positive medium-frequency CARs. Similar heterogeneity has been reported in other studies of biopharmaceutical events. Singh et al. found that even among clinical trial result events, abnormal returns varied with characteristics such as firm type, trial phase, and disease area, while these attributes remained insufficient to explain the full variation in excess returns [9]. The cross-event comparison in this study further shows that even policies employing similar instruments can differ in the breadth and distribution of sectoral responses, as illustrated by the two centralized procurement events. Thus, within the events examined here, policy attributes provide useful institutional context for interpreting market reactions but do not offer a stable basis for classifying the direction or sectoral distribution of abnormal returns.

Whether an aggregate industry index adequately captures sub-sector responses depends on the degree of consistency across constituent industries within a given event. Kapar et al., comparing industry and sub-industry indices, found that market reactions to the same information event can differ markedly across levels of aggregation, such that aggregate industry results may attenuate differences among underlying sub-sectors [25]. A similar pattern is observed in this study, although differences between the first-level pharmaceutical index and its sub-sector indices were not equally pronounced across all events. During the review and approval system reform, the Shenwan first-level Pharmaceutical and Biotechnology Index and all six pharmaceutical sub-sector indices showed medium-frequency CARs in the same direction, allowing the aggregate index to provide a reasonable summary of the common response. By contrast, clearer directional divergence across pharmaceutical sub-sectors emerged during the clinical trial data self-inspection and verification event, the centralized procurement of high-value medical consumables, and the DRG/DIP payment reform. The comparison between the two centralized procurement events illustrates this distinction particularly clearly. Centralized drug procurement was associated with broad negative accumulation across sub-sectors, whereas centralized procurement of high-value medical consumables, although also generally negative, showed a much stronger negative response in Medical Devices, while Pharmaceutical Distribution moved in the opposite direction. The aggregate index therefore neither necessarily represents nor necessarily obscures sub-sector responses. When sub-sectors move in broadly the same direction, it can provide a useful summary of the common market response. When internal responses diverge, however, the aggregate index reflects a weighted overall outcome rather than a reaction shared by all constituent sub-sectors.

The multiscale analysis further shows that abnormal-return patterns observed for the same event are not always consistent across statistical scales. Previous studies have combined EEMD with event analysis to examine how the same market shock is reflected in different frequency components, suggesting that single-scale analysis may overlook some multiscale features [26]. In this study, Chemical Pharmaceuticals, Traditional Chinese Medicine, and Medical Devices during the DRG/DIP payment reform, as well as Pharmaceutical Distribution during the clinical trial data self-inspection and verification event, repeatedly showed opposite directions of high- and medium-frequency CARs across multiple event windows. These results suggest that examining a single return scale may sometimes provide an incomplete representation of the abnormal-return structure around an event. Because this study does not conduct formal tests of differences across scales, these findings should be interpreted as descriptive comparisons. Moreover, the high- and medium-frequency components are, in the first instance, statistical scales derived from EEMD decomposition and should not be directly equated with investors’ short- and medium-term assessments. Within this interpretive boundary, the value of multiscale analysis lies in showing that the direction of abnormal returns observed at one scale does not necessarily extend to other statistical scales, rather than in assigning specific behavioral interpretations to different frequency components.

CARs over wider event windows should likewise not be interpreted directly as post-announcement market reactions. Event 6 provides a clear example. Its medium-frequency CARs over wider windows were generally negative, yet the negative ARs for most indices occurred mainly before the event date. These ARs subsequently increased and turned positive after the announcement. A negative CAR over a wider window therefore does not imply that abnormal returns after the announcement were also negative. Event studies typically measure changes in security prices around a specific information event, but the interpretation of multi-day event windows becomes more complicated when the exact event date is uncertain or when price adjustment extends beyond a single trading day [5,27]. Cho et al., in a large-scale study of biopharmaceutical news, similarly identified abnormal returns before the news release date for some categories of events [11]. This study cannot determine whether the negative ARs observed before Event 6 reflected policy expectations, previously available information, or other contemporaneous factors, and there is no evidence to interpret them as information leakage. What can be established is that CAR represents the net accumulation of abnormal returns over the entire event window, whereas daily ARs retain information on the temporal path through which that cumulative outcome develops. When the direction of ARs changes clearly within an event window, relying solely on a wider-window CAR to characterize the post-announcement market response may therefore provide an incomplete picture.

Taken together, this study does not seek to rank the six pharmaceutical policies according to simple positive or negative market reactions. Rather, the findings show that abnormal returns around policy events cannot be adequately summarized by policy orientation, aggregate industry CARs, or a single statistical scale. Similar policy instruments may be associated with different sectoral responses, an aggregate index may not represent the common movement of its constituent sub-sectors, and the same event may produce different patterns across statistical scales and event windows. These comparisons nevertheless have clear limitations. The six events occurred in different years and market environments, and no formal tests of differences across events or scales were conducted. The official policy release date may also not coincide with the point at which information first entered the market, while industry indices cannot identify actual policy exposure at the firm level. In addition, EEMD was applied ex post to the full-sample return series, so its frequency components should primarily be interpreted as statistical scales. The simultaneous comparison of multiple events, industries, scales, and event windows also raises a multiple-comparison issue. Accordingly, the analysis emphasizes recurring descriptive patterns that are corroborated by daily AR trajectories rather than isolated findings of statistical significance. The ARs and CARs reported here should therefore be interpreted as price changes relative to the market benchmark around specific policy information events, rather than as direct measures of the long-term effects or relative merits of the policies.

## Conclusion

### Main findings

This study combines EEMD-based multiscale reconstruction with an event-study approach to compare abnormal returns around six pharmaceutical policy events in China using the Shenwan first-level Pharmaceutical and Biotechnology Index and six pharmaceutical sub-sector indices. The results show that, within the events, industry indices, and event windows examined, policy objectives or policy-instrument attributes did not exhibit a consistent correspondence with the observed abnormal-return patterns. Medium-frequency CARs were generally negative for both centralized procurement events, but centralized drug procurement showed broader negative accumulation across sub-sectors, whereas centralized procurement of high-value medical consumables exhibited greater sectoral differentiation, with Medical Devices showing the largest negative magnitude. Although the clinical trial data self-inspection and verification event and the review and approval system reform both involved drug R&D and registration regulation, their abnormal-return structures also differed markedly. Thus, within the events examined, policy attributes provide useful institutional context for describing different policy events but do not show a stable empirical correspondence with the direction or sectoral distribution of abnormal returns.

Further comparisons show that different industry-index levels, return scales, and event windows reveal different aspects of abnormal-return patterns. Whether an aggregate industry index adequately summarizes sub-sector responses depends on the degree of consistency across pharmaceutical sub-sectors within a given event. When sub-sector responses diverge markedly, the first-level pharmaceutical index cannot separately reflect the underlying sub-sector differences. For some events and sub-sectors, high- and medium-frequency CARs showed opposite directions across multiple event windows, while the cumulative direction of CARs over wider windows could also differ from the trajectory of daily ARs following the announcement. Taken together, these findings suggest that generalizations based on policy categories, an aggregate industry index, or a single event window may overlook important differences in abnormal returns around policy events. Sensitivity analyses further showed that the main medium-frequency CAR patterns across events and sub-sectors remained broadly similar under alternative decomposition specifications.

### Research implications

The ARs and CARs observed in this study reflect price changes relative to the market benchmark around specific policy information events and should not be interpreted directly as measures of the long-term economic effects of policies, their effects on industry development, or their relative merits. Analysis of market responses to pharmaceutical policy events should likewise not rely solely on policy orientation, an aggregate industry index, or a single event window. Comparisons across sub-sectors, multiscale analysis, and the joint examination of daily ARs and CARs can provide information that may not be fully captured by a single aggregate index or a single cumulative-return measure. At the same time, the high- and medium-frequency components are, in the first instance, return components derived from statistical decomposition and should not be interpreted directly as representing different investment horizons or independent policy effects.

### Limitations and future directions

The conclusions of this study are primarily limited to the six policy events, industry indices, and event windows examined. Because the events occurred under different market conditions, no formal tests of differences across events or statistical scales were conducted, and industry indices cannot identify actual policy exposure at the firm level. In addition, the official policy release date may not coincide with the point at which information first entered the market, while contemporaneous macroeconomic and industry-specific information may also have affected returns within the event windows. The simultaneous comparison of multiple events, industries, scales, and windows also raises a multiple-comparison issue. Accordingly, the main interpretations in this study place greater emphasis on recurring descriptive patterns that are corroborated by daily AR trajectories than on isolated findings of statistical significance. Future research could expand the sample of policy events, incorporate firm-level measures of policy exposure and operating characteristics, and further distinguish among the timing of policy expectation formation, information disclosure, and official policy release. Such extensions would help assess whether the differences observed here across industry levels, statistical scales, and event window are generalizable beyond the events examined in this study.

## Supporting information

S1 Table EEMD decomposition metrics by industry. (DOCX)

S1 Data Raw data used in the empirical analysis. (ZIP)
