# Supplementary material for: Heterogeneous stock market responses to pharmaceutical policy events across subsectors and time scales: Evidence from China
Source: PLoS One. 2026 Sep 25;21(9):e0353989. doi: 10.1371/journal.pone.0353989 (PMC13614644; doi:10.1371/journal.pone.0353989)
Supplement: Supplementary file 1 [file pone.0353989.s001.docx]

# Supporting information

**Table S1. EEMD Decomposition Metrics by Industry**

| **industry** | **Component** | **AvgPeriod** | **Mean** | **Std** | **Variance** | **VarRatioPct** |
| --- | --- | --- | --- | --- | --- | --- |
| CSI 300 | IMF1 | 2.902145474 | -2.02E-05 | 0.010402495 | 0.000108212 | 57.80095465 |
|  | IMF2 | 6.032644178 | 1.10E-05 | 0.005633334 | 3.17E-05 | 16.95082881 |
|  | IMF3 | 11.02186879 | 4.71E-05 | 0.004067356 | 1.65E-05 | 8.836580794 |
|  | IMF4 | 21.48638132 | 2.52E-05 | 0.002805087 | 7.87E-06 | 4.202933856 |
|  | IMF5 | 41 | -1.22E-05 | 0.001962433 | 3.85E-06 | 2.057073696 |
|  | IMF6 | 84.15384615 | -1.82E-05 | 0.001582415 | 2.50E-06 | 1.337521215 |
|  | IMF7 | 149.9411765 | -1.51E-05 | 0.000934345 | 8.73E-07 | 0.466310148 |
|  | IMF8 | 382.7692308 | 4.14E-05 | 0.000687852 | 4.73E-07 | 0.252725936 |
|  | IMF9 | 619.4285714 | 2.42E-05 | 0.000575576 | 3.31E-07 | 0.176955968 |
|  | IMF10 | 1338.666667 | 3.05E-05 | 0.000408715 | 1.67E-07 | 0.089228103 |
|  | IMF11 | 65535 | 0.000217129 | 0.000141752 | 2.01E-08 | 0.010732956 |
|  | IMF12 | 65535 | 4.06E-05 | 2.21E-05 | 4.90E-10 | 0.00026155 |
|  | Residual |  | 3.20E-06 | 2.16E-06 | 4.65E-12 | 2.48E-06 |
| Pharma-Bio | IMF1 | 2.986006459 | 7.08E-06 | 0.012526307 | 0.000156908 | 57.30133452 |
|  | IMF2 | 6.247747748 | 5.19E-05 | 0.006457162 | 4.17E-05 | 15.22656706 |
|  | IMF3 | 11.13078471 | 2.96E-05 | 0.004615764 | 2.13E-05 | 7.780468719 |
|  | IMF4 | 21.96 | 2.61E-05 | 0.003314354 | 1.10E-05 | 4.011588241 |
|  | IMF5 | 42.41538462 | -1.04E-05 | 0.002383058 | 5.68E-06 | 2.073900763 |
|  | IMF6 | 83.36363636 | 1.03E-05 | 0.002054557 | 4.22E-06 | 1.541541192 |
|  | IMF7 | 156.8484848 | -1.64E-05 | 0.001019666 | 1.04E-06 | 0.37969438 |
|  | IMF8 | 320 | 1.07E-05 | 0.000609619 | 3.72E-07 | 0.135717235 |
|  | IMF9 | 636.5714286 | 4.00E-05 | 0.000544425 | 2.96E-07 | 0.108241714 |
|  | IMF10 | 1289.333333 | 4.41E-05 | 0.000346585 | 1.20E-07 | 0.043866918 |
|  | IMF11 | 2038 | 0.000143269 | 0.000238443 | 5.69E-08 | 0.020762801 |
|  | IMF12 | 1026 | 2.09E-05 | 1.98E-05 | 3.92E-10 | 0.000143055 |
|  | Residual |  | 1.49E-06 | 5.10E-06 | 2.61E-11 | 9.51E-06 |
| Chem Pharm | IMF1 | 2.947927736 | -0.000125466 | 0.012767253 | 0.000163003 | 57.82579987 |
|  | IMF2 | 6.2760181 | 6.37E-05 | 0.007004595 | 4.91E-05 | 17.40574875 |
|  | IMF3 | 11.35934292 | -8.62E-06 | 0.004855905 | 2.36E-05 | 8.365021084 |
|  | IMF4 | 23.92173913 | 3.28E-05 | 0.003247481 | 1.05E-05 | 3.741278588 |
|  | IMF5 | 41.14925373 | 1.44E-05 | 0.002389021 | 5.71E-06 | 2.024726873 |
|  | IMF6 | 98.32727273 | -2.88E-06 | 0.001884881 | 3.55E-06 | 1.260360195 |
|  | IMF7 | 185.4482759 | -1.63E-05 | 0.000901809 | 8.13E-07 | 0.288506875 |
|  | IMF8 | 319.875 | -6.71E-06 | 0.000588608 | 3.46E-07 | 0.122907712 |
|  | IMF9 | 634.5714286 | 3.88E-05 | 0.000548303 | 3.01E-07 | 0.106651787 |
|  | IMF10 | 1262 | 0.000103185 | 0.000410303 | 1.68E-07 | 0.05972212 |
|  | IMF11 | 65535 | 0.000397298 | 0.000172906 | 2.99E-08 | 0.01060584 |
|  | Residual |  | 2.15E-05 | 7.61E-06 | 5.80E-11 | 2.06E-05 |
| TCM | IMF1 | 2.907756813 | -1.11E-05 | 0.012180431 | 0.000148363 | 57.3452121 |
|  | IMF2 | 5.978448276 | 7.83E-05 | 0.006286429 | 3.95E-05 | 15.27495082 |
|  | IMF3 | 11.60251046 | 4.78E-06 | 0.004622582 | 2.14E-05 | 8.259260998 |
|  | IMF4 | 22.63114754 | 6.94E-06 | 0.003284909 | 1.08E-05 | 4.170790907 |
|  | IMF5 | 43.34920635 | 6.55E-06 | 0.002182338 | 4.76E-06 | 1.840839941 |
|  | IMF6 | 89.04918033 | -2.02E-05 | 0.001771667 | 3.14E-06 | 1.21321071 |
|  | IMF7 | 155.8787879 | -2.08E-05 | 0.000966344 | 9.34E-07 | 0.360940397 |
|  | IMF8 | 313.4117647 | 1.85E-05 | 0.000608908 | 3.71E-07 | 0.143309414 |
|  | IMF9 | 618.75 | 1.93E-05 | 0.000325405 | 1.06E-07 | 0.040927975 |
|  | IMF10 | 1342 | 4.65E-05 | 0.00034577 | 1.20E-07 | 0.046211191 |
|  | IMF11 | 1780 | 5.69E-05 | 0.000168266 | 2.83E-08 | 0.010943767 |
|  | IMF12 | 65535 | 1.29E-05 | 4.29E-05 | 1.84E-09 | 0.00071007 |
|  | Residual |  | 8.27E-07 | 1.06E-06 | 1.13E-12 | 4.36E-07 |
| Biotech | IMF1 | 2.904712042 | 4.68E-05 | 0.013473321 | 0.00018153 | 54.98036538 |
|  | IMF2 | 6.070021882 | 1.28E-05 | 0.007661416 | 5.87E-05 | 17.77773134 |
|  | IMF3 | 11.5010395 | 2.64E-05 | 0.00538555 | 2.90E-05 | 8.784527868 |
|  | IMF4 | 22.24193548 | 2.67E-05 | 0.003732829 | 1.39E-05 | 4.220213607 |
|  | IMF5 | 42.43076923 | -3.03E-06 | 0.002999058 | 8.99E-06 | 2.724131551 |
|  | IMF6 | 80.70588235 | 1.17E-05 | 0.002104622 | 4.43E-06 | 1.34154948 |
|  | IMF7 | 166.0645161 | -7.90E-06 | 0.001111107 | 1.23E-06 | 0.373912256 |
|  | IMF8 | 288.3333333 | 3.46E-06 | 0.000670084 | 4.49E-07 | 0.135993154 |
|  | IMF9 | 665.4285714 | 1.88E-05 | 0.000600947 | 3.61E-07 | 0.109378004 |
|  | IMF10 | 1290.666667 | 1.71E-05 | 0.000368876 | 1.36E-07 | 0.041211627 |
|  | IMF11 | 65535 | 0.000158719 | 0.000341301 | 1.16E-07 | 0.035280389 |
|  | IMF12 | 65535 | 3.32E-05 | 5.65E-05 | 3.19E-09 | 0.00096569 |
|  | Residual |  | 1.99E-06 | 2.57E-06 | 6.62E-12 | 2.00E-06 |
| Pharma Dist. | IMF1 | 2.926160338 | -2.11E-06 | 0.012360295 | 0.000152777 | 56.87508759 |
|  | IMF2 | 5.948442535 | -1.35E-05 | 0.006894696 | 4.75E-05 | 17.69679455 |
|  | IMF3 | 11.26829268 | -4.16E-05 | 0.005068909 | 2.57E-05 | 9.565185972 |
|  | IMF4 | 20.82264151 | 1.59E-05 | 0.003447593 | 1.19E-05 | 4.424826719 |
|  | IMF5 | 42.953125 | -1.52E-05 | 0.0024183 | 5.85E-06 | 2.17713235 |
|  | IMF6 | 85.65625 | -1.02E-05 | 0.001723139 | 2.97E-06 | 1.105363214 |
|  | IMF7 | 203.6923077 | -1.14E-05 | 0.00086792 | 7.53E-07 | 0.280429333 |
|  | IMF8 | 304.9411765 | 1.55E-05 | 0.000616844 | 3.80E-07 | 0.141649544 |
|  | IMF9 | 657.4285714 | 2.22E-05 | 0.000580504 | 3.37E-07 | 0.125451289 |
|  | IMF10 | 1104.666667 | 4.55E-05 | 0.00030194 | 9.12E-08 | 0.033939422 |
|  | IMF11 | 1148 | 0.00014177 | 0.000201749 | 4.07E-08 | 0.015152621 |
|  | IMF12 | 65535 | 3.11E-05 | 3.20E-05 | 1.02E-09 | 0.000380582 |
|  | Residual |  | 1.00E-06 | 5.48E-07 | 3.00E-13 | 1.12E-07 |
| Med Devices | IMF1 | 2.957356077 | 9.48E-06 | 0.014473608 | 0.000209485 | 56.28011891 |
|  | IMF2 | 5.997835498 | -7.73E-06 | 0.007896301 | 6.24E-05 | 16.75131092 |
|  | IMF3 | 11.42975207 | 4.83E-05 | 0.00610818 | 3.73E-05 | 10.02363108 |
|  | IMF4 | 21.45882353 | 6.41E-05 | 0.003792996 | 1.44E-05 | 3.865148764 |
|  | IMF5 | 42.16923077 | 9.30E-06 | 0.002946183 | 8.68E-06 | 2.33195787 |
|  | IMF6 | 88.2295082 | 1.45E-05 | 0.002418048 | 5.85E-06 | 1.570837926 |
|  | IMF7 | 157.6470588 | -1.75E-05 | 0.001173533 | 1.38E-06 | 0.369991549 |
|  | IMF8 | 292 | -1.92E-05 | 0.000870935 | 7.59E-07 | 0.203785501 |
|  | IMF9 | 633.7142857 | 4.17E-05 | 0.000623906 | 3.89E-07 | 0.104577722 |
|  | IMF10 | 1460.666667 | 2.46E-05 | 0.000615385 | 3.79E-07 | 0.101740755 |
|  | IMF11 | 65535 | 0.000115339 | 0.00016826 | 2.83E-08 | 0.007606147 |
|  | Residual |  | 3.96E-05 | 2.66E-05 | 7.08E-10 | 0.000190305 |
| Healthcare | IMF1 | 2.913865546 | -1.93E-05 | 0.015888299 | 0.000252438 | 54.43367757 |
|  | IMF2 | 6.079034029 | 3.36E-05 | 0.008855716 | 7.84E-05 | 16.91064546 |
|  | IMF3 | 11.86295503 | 1.82E-05 | 0.006395139 | 4.09E-05 | 8.818867357 |
|  | IMF4 | 22.19354839 | 3.13E-05 | 0.004501277 | 2.03E-05 | 4.369022673 |
|  | IMF5 | 43.24409449 | 3.90E-06 | 0.003323407 | 1.10E-05 | 2.381661486 |
|  | IMF6 | 85.3015873 | 4.20E-05 | 0.002680438 | 7.18E-06 | 1.549259988 |
|  | IMF7 | 179.5172414 | 8.56E-07 | 0.001425001 | 2.03E-06 | 0.437867878 |
|  | IMF8 | 380.1428571 | 2.26E-05 | 0.001099908 | 1.21E-06 | 0.260870804 |
|  | IMF9 | 621.4285714 | 5.38E-05 | 0.000750796 | 5.64E-07 | 0.121550567 |
|  | IMF10 | 1420 | 7.29E-05 | 0.00058133 | 3.38E-07 | 0.072871517 |
|  | IMF11 | 65535 | 0.0002954 | 0.000233222 | 5.44E-08 | 0.011728803 |
|  | Residual |  | 1.12E-05 | 6.19E-06 | 3.83E-11 | 8.26E-06 |
